# Post-obligation physician retention among Jichi Medical University graduates in Okinawa prefecture, Japan: a descriptive comparative study of the Kyushu region

**DOI:** 10.1186/s12875-026-03521-0

**Published:** 2026-08-13

**Authors:** Kohta Oyama, Yoshikazu Kawazuma, Ryusuke Ae, Hiroshi Noda, Kunihiko Matsui

**Affiliations:** 1 Department of Health and Welfare, Health Bureau, Medical Policy Division, Kumamoto Prefectural Office, Kumamoto, Japan; 2 Okinawa Community Medicine Support Centre, University of the Ryukyus Hospital, Okinawa, Japan; 3 Division of Public Health, Centre for Community Medicine, Jichi Medical University, Tochigi, Japan; 4 Department of Surgery, Saitama Medical Centre, Jichi Medical University, Saitama, Japan; 5 Department of General Medicine and Primary Care, Kumamoto University Hospital, Kumamoto, Japan

**Keywords:** Physicians, Personnel retention, Rural health services, Islands, Japan, Health workforce, Medically underserved area

## Abstract

**Background:**

Global strategies to address physician maldistribution include recruiting students with rural backgrounds, community-based training, bonded service, financial incentives, and personal and professional support. However, retention after obligatory service remains variable. Jichi Medical University (JMU) in Japan was established to address physician shortages in rural areas through a scholarship loan programme requiring graduates to complete nine years of service in their home prefectures. This study compares post-obligation retention patterns across the Kyushu prefectures and uses Okinawa as a context for secondary-source-based interpretation of why retention may differ across prefectures operating under a shared obligation framework.

**Methods:**

This descriptive comparative study combined a cross-sectional analysis of retention data from eight Kyushu prefectures using the 2024 JMU Graduate Directory with a secondary-source-based contextual interpretation of Okinawa. Among 641 graduates who had completed medical school at least 10 years previously, retention was operationally defined as practising within the home prefecture on 1 July 2024. Quantitative analyses compared retention rates, prefectural capital concentration, and the ecological correlation between inhabited-island count and retention. The contextual interpretation drew on a review-derived framework linking geographical access conditions, institutional support and training arrangements, and sociocultural embeddedness to physician-retention factors described in previous reviews.

**Results:**

Okinawa demonstrated the highest retention rate (84.2%) among the eight prefectures, exceeding Kagoshima (67.8%) and Saga (44.2%). In sensitivity analysis, excluding unknown workplace cases, Okinawa remained the highest at 90.1%, and the overall pattern was similar. No clear linear association was observed between inhabited-island count and retention. Descriptive comparison and secondary-source-based contextual interpretation identified three hypothesis-generating domains that may help interpret Okinawa’s pattern: (1) comprehensive structured training and support, (2) distributed healthcare infrastructure and placement arrangements, and (3) cultural cohesion characterised by communal reciprocity (*yuimaru*) and strong regional identity.

**Conclusions:**

Okinawa’s comparatively high physician retention may be interpreted in relation to a combination of structured training and support systems, distributed healthcare infrastructure, and cultural cohesion. Because the contextual interpretation was based on secondary sources, these inferences should be regarded as hypothesis-generating rather than explanatory. Nevertheless, comparison across prefectures within a shared JMU obligation framework highlights how implementation context may be relevant to post-obligation retention and identifies priorities for future empirical research.

**Supplementary Information:**

The online version contains supplementary material available at https://doi.org/10.1186/s12875-026-03521-0.

## Background

The geographic maldistribution of physicians is a global challenge, with rural and remote areas experiencing critical shortages of medical professionals. International evidence suggests that physician retention is shaped by multiple interacting determinants, including rural background, community-based education and rural rotations, bonded service and financial incentives, professional support and mentorship, working and living conditions, and community integration [1–7]. In Japan, Jichi Medical University (JMU) was established in 1972 to train physicians specifically for practice in medically underserved regions. As the only nationwide physician training programme jointly operated by all 47 prefectures [8], JMU selects students from each prefecture, who return to their home regions after graduation.

JMU’s distinctive system requires graduates to serve in their home prefectures for nine years after graduation (the obligation period) [9, 10]. Its scholarship loan programme covers tuition and educational expenses [11, 12] and is forgiven upon fulfilment of the obligation. During this period, graduates spend four to five years at remote or rural medical institutions as public servants, with assignments determined by gubernatorial instruction. Since graduating its first class in 1978, JMU has trained over 4,000 physicians, who form the backbone of Japan’s rural and remote island healthcare [13].

The national average prefectural retention rate among JMU graduates was 69.8%, with rates varying substantially across prefectures (45.5–93.3%) [9]. This study focused on Okinawa prefecture, which demonstrates the highest retention rate among the Kyushu prefectures (84.2%). The Kyushu region provides a suitable comparative context: all eight prefectures face similar rural healthcare challenges yet exhibit divergent retention outcomes (44.2–84.2%). Okinawa comprises 160 islands, including 38 inhabited remote islands located approximately 600 km from the Kyushu mainland, suggesting that factors beyond geography may be relevant. Existing literature has described broad determinants of physician retention, including support systems, rural training, working conditions, and community integration [1–7, 14, 15]. However, it remains unclear why retention varies so markedly across prefectures operating under the same JMU obligation framework. This variation within the programme offers a policy-relevant comparative setting because the basic bonded-service scheme is shared across prefectures, allowing attention to shift toward differences in implementation context. Accordingly, this study aimed to compare post-obligation retention patterns across the Kyushu prefectures and to use Okinawa as a contextual case to generate hypotheses about how geographical access conditions, institutional support and training arrangements, and sociocultural embeddedness may help interpret especially high retention.

## Methods

### Study design and setting

This was a descriptive comparative study comprising (1) a cross-sectional comparison of post-obligation physician retention among JMU graduates across eight Kyushu prefectures and (2) a secondary-source-based contextual interpretation of Okinawa. The analytical framework was derived deductively from prior systematic and narrative reviews of physician retention factors [1–7, 14, 15]. For the purposes of this study, review-identified determinants were grouped into three context-level domains that could be examined across prefectures using directory data and secondary sources: geographical access conditions, institutional support and training arrangements, and sociocultural embeddedness. Because the scholarship obligation system and basic bonded-service requirement were common across all prefectures, they were treated as shared background conditions rather than differentiating variables. The directory data constituted the empirical component, whereas the Okinawa component was used to support contextual interpretation, not to provide a formal qualitative case analysis or to empirically test explanatory relationships. Accordingly, the contextual interpretation should be understood as hypothesis-generating.

### Data sources and participants

Data on JMU graduates in the eight prefectures were obtained from the 2024 JMU Graduate Directory [16], which listed current workplace locations (prefecture, municipality, and facility type) as of 1 July 2024. The study included all graduates who had completed medical school at least 10 years previously (graduated in 2014 or earlier; *n* = 641). This threshold ensured that virtually all eligible graduates would have completed the nine-year obligation period. Geographical data on the number of inhabited islands per prefecture were obtained from government reports [17]. Information on support programmes, training pathways, and cultural factors relevant to Okinawa was compiled through a purposive review of published articles, programme descriptions and publicly available reports [18–22].

### Retention rate

Retention rate was defined as the percentage of eligible graduates practising in their home prefecture as of 1 July 2024. Home prefecture was defined as the prefecture from which a graduate was originally admitted to JMU. This definition differed from previous longitudinal definitions of retention. However, it was chosen because the directory provided a cross-sectional snapshot of current practice locations rather than the duration of post-obligation service [9]. Hence, the measure was interpreted as current in-prefecture practice among graduates who had completed the obligation period, instead of a direct measure of voluntary long-term commitment.

Since JMU recruits two to three students per prefecture annually through a standardised selection process [9], the number of graduates per prefecture is approximately equal across the study sample (range: 75–87). This structurally reduced the likelihood that Okinawa’s higher retention rate was the result of a disproportionately large number of Okinawa-origin students in the sample. Physicians whose current workplace could not be determined (*n* = 42, 6.6%) were conservatively classified as ‘not retained’ for the primary analysis. A sensitivity analysis, excluding these cases, was conducted and is presented in Supplementary Table S2. Retention rates were summarised using counts and proportions (Microsoft Excel 2022).

### Healthcare access barriers analysis

The number of inhabited islands in each prefecture was used as a proxy indicator for geographical barriers to medical facility access. Pearson’s correlation coefficient (r) and its 95% confidence interval (CI) assessed the ecological relationship between island count and prefecture-level retention rates. Since only eight prefectures were included and more direct indicators of access (e.g. travel time, transportation frequency, weather-related disruption, and inter-island connectivity) were unavailable, this analysis was exploratory and intended to assess whether a linear pattern was evident rather than estimating a causal effect of geography. To examine physician distribution patterns, prefectural capital concentration rates were calculated as the proportion of retained JMU graduates working in the prefectural capital city. Furthermore, public versus private institution employment was compared across prefectures.

### Okinawa’s medical care delivery system

A literature-informed, secondary-source-based contextual interpretation of the selected documents [18–22] and publicly available data on prefectural healthcare infrastructure was undertaken to describe characteristics of Okinawa’s medical care delivery system that may help interpret the observed retention pattern. Sources were purposively selected if they described supervision, rotation programmes, substitute physician support, remote-island healthcare infrastructure, community integration, or Okinawa’s cultural and historical context. Extracted information was organised deductively according to the pre-specified geographical, institutional, and cultural domains. Since this contextual component was intended to interpret the empirical retention patterns rather than generate an independent qualitative theory or test causal mechanisms directly, formal inter-rater reliability testing was not undertaken.

### Ethical considerations

The JMU Clinical Research Ethics Committee approved this study (Approval ID: 25–041) and waived the requirement for informed consent because the study used existing directory data analysed at the aggregate level. An opt-out notice was posted on the university website (https://www.jichi.ac.jp/dph/wp-content/uploads/2025/07/20250729_joho_okinawa.pdf) 30 days before analysis, and no exclusion requests were received. All data were de-identified and presented in aggregate form.

## Results

### Comparison of retention rates

**Fig. 1:**
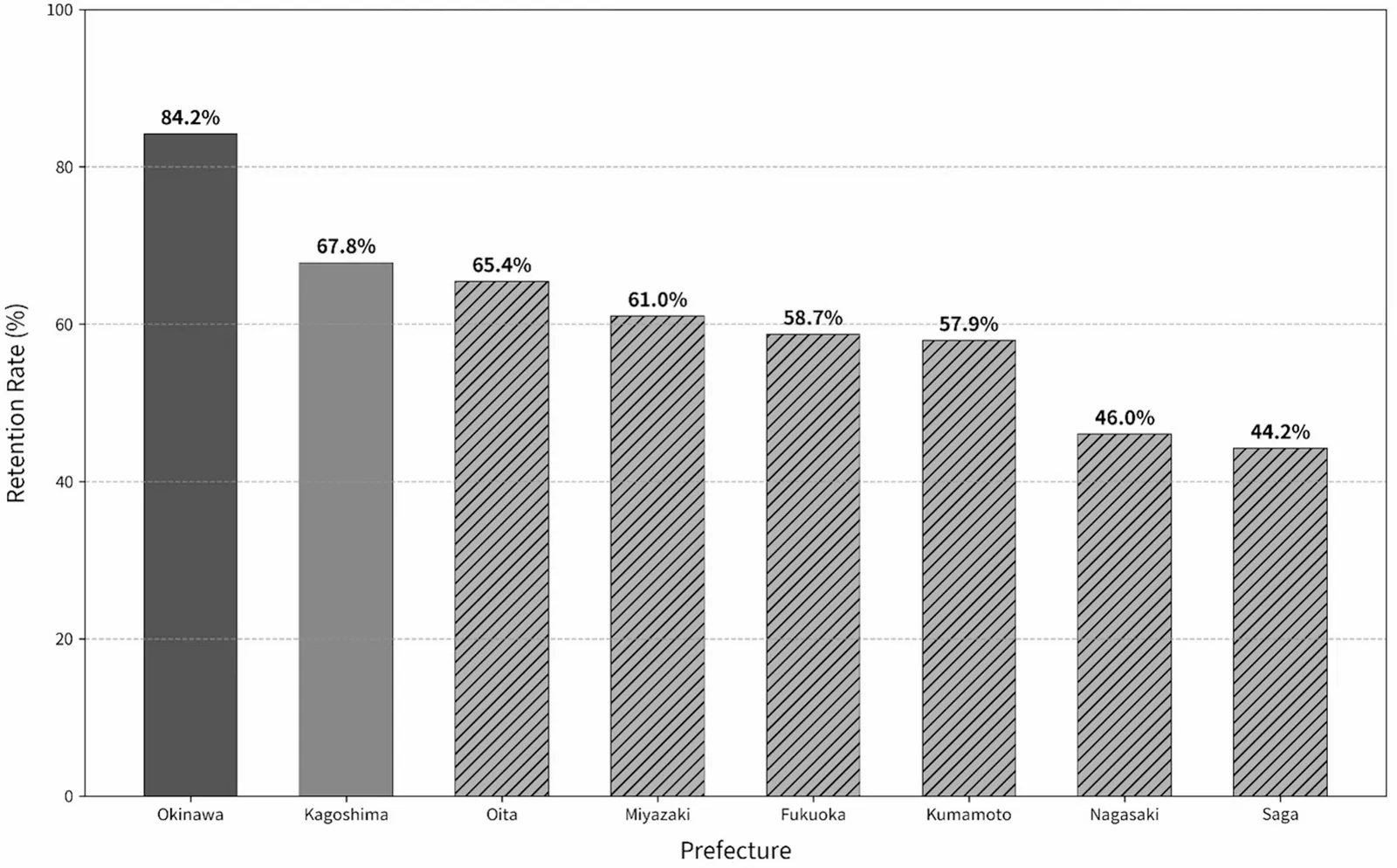
JMU graduates’ post-obligation physician retention rates in eight Kyushu prefectures (2024). This bar left t illustrates the percentage of JMU graduates who continued practising in their home prefecture as of 1 July 2024, among those who graduated in 2014 or earlier (≥ 10 years post-graduation). Prefectures are ordered by retention rate, from highest to lowest. Okinawa prefecture (solid bar) demonstrates the highest retention rate (84.2%), substantially exceeding all other prefectures. The hatched bars represent the seven comparison prefectures in Kyushu. Retention rates range from 44.2% (Saga) to 84.2% (Okinawa), with an overall retention rate of 60.5%. Data source: JMU Medical School Graduate Directory 2024

The analysis included 641 JMU graduates from the eight study prefectures, who graduated in 2014 or earlier (≥ 10 years post-graduation) and were expected to have completed the nine-year obligation. Retention rates ranged from 44.2% (Saga) to 84.2% (Okinawa), with an overall retention rate of 60.5%. Okinawa exhibited the highest retention rate (84.2%), while the lowest rates were observed in Saga (44.2%) and Nagasaki (46.0%) (Table 1; Fig. 1).

**Table 1:** JMU graduates’ post-obligation physician retention rates in eight Kyushu prefectures (1 July 2024)

Prefecture	Retention Rate (%)	Physicians Working Within the Prefecture	Physicians Working Outside the Prefecture	Capital City Concentration Rate (%)	Public Hospital/Clinic Physicians	Private Hospital Physicians	Number of Inhabited Islands
Okinawa	84.2	64	12 (5)	17.2	32	24	38
Kagoshima	67.8	59	28 (2)	44.1	18	24	20
Oita	65.4	53	28 (10)	47.2	12	27	7
Miyazaki	61	50	32 (2)	50	22	12	3
Fukuoka	58.7	44	31 (8)	13.6	17	17	8
Kumamoto	57.9	44	32 (5)	61.4	10	22	6
Nagasaki	46	40	47 (6)	15.0	22	8	51
Saga	44.2	34	43 (4)	52.9	11	16	7

Source: Ministry of Land, Infrastructure, Transport and Tourism April 2025 [17]

Data compiled from the JMU Medical School Graduate Directory [16].Physicians employed at unidentified institutions were counted among those practising outside the prefecture

In sensitivity analysis, excluding 42 graduates with unknown practice locations, Okinawa remained the highest-retention prefecture (90.1%). Furthermore, the overall pattern remained similar despite prefecture-specific changes of + 1.5 to + 9.2% points (Supplementary Table S2). These findings suggest that the conservative classification of unknown cases as non-retained did not materially alter the main interpretation of Okinawa’s distinctive position.

### Analysis of healthcare access barriers and retention patterns

**Fig. 2:**
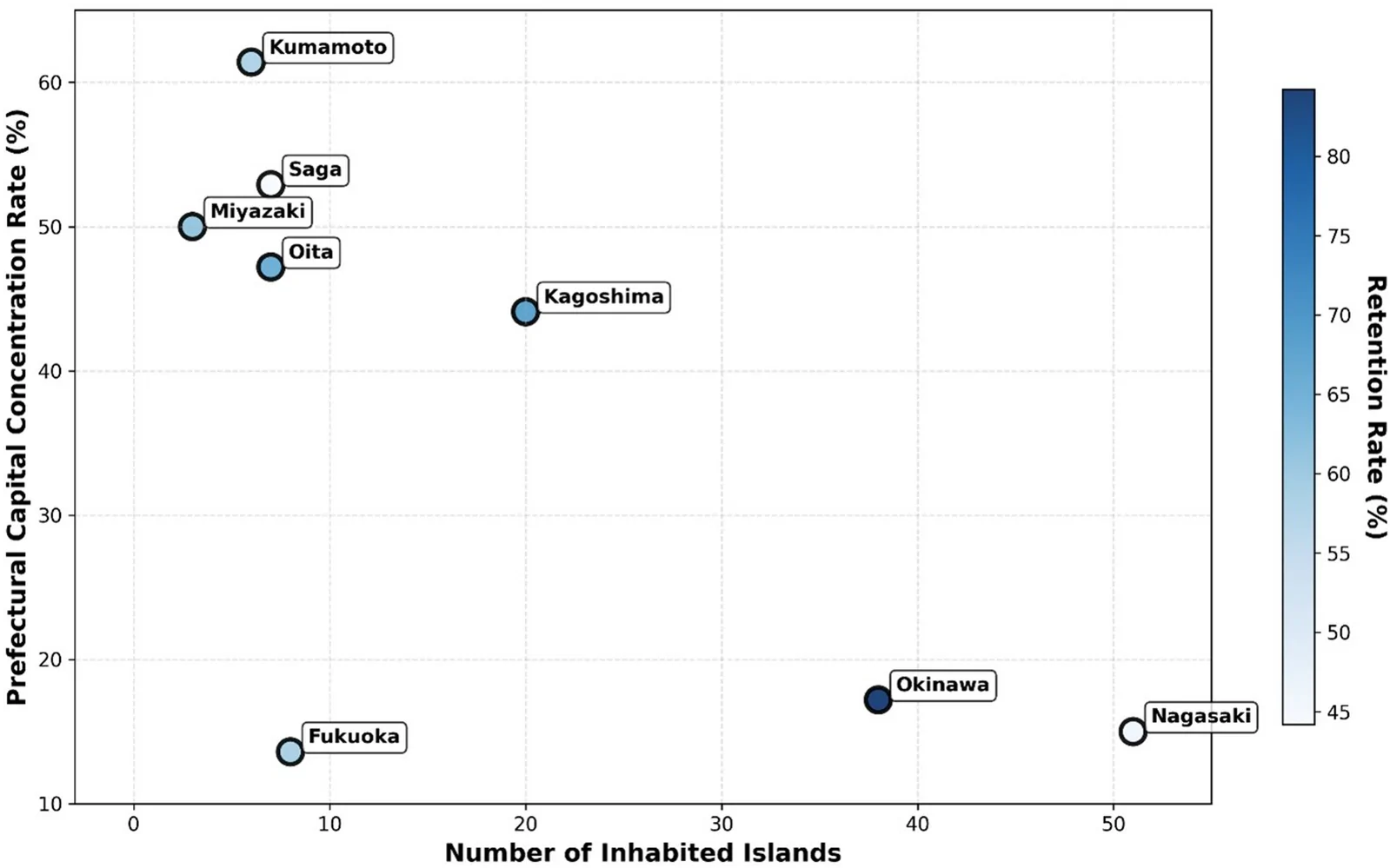
Ecological comparison of inhabited-island count, prefectural capital concentration, and physician retention across the eight Kyushu prefectures. This scatter plot shows the relationship between the number of inhabited islands (x-axis) and prefectural capital concentration rate (y-axis), with colour indicating physician retention rate (darker blue = higher retention). No clear linear association was observed between inhabited-island count and retention rate (Pearson’s r = 0.146, 95% CI = -0.623 to 0.771; p = 0.730). These findings should be interpreted cautiously because the analysis was based on prefecture-level ecological comparison with a small sample size. Data sources: JMU Medical School Graduate Directory 2024; Ministry of Land, Infrastructure, Transport and Tourism (April 2025)

Although Okinawa prefecture has 38 inhabited remote islands—the second highest among the eight study prefectures—no clear linear association was observed between the number of inhabited islands and retention rates (Fig. 2). Statistical analysis supported this observation, with a Pearson’s *r* = 0.146 (95% CI = -0.623 to 0.771, *p* = 0.730). Given the small number of prefectures and the ecological nature of the comparison, this finding should be interpreted cautiously. Rather than demonstrating a geographical effect, it indicates that the inhabited-island count alone was insufficient to explain the marked between-prefecture retention variation.

Moreover, prefectural capital concentration varied substantially across prefectures. In Okinawa, 17.2% of retained JMU graduates worked in the prefectural capital. This indicated relatively low concentration in Naha compared with several other prefectures (Kumamoto = 61.4%, Saga = 52.9%, Miyazaki = 50.0%, Oita = 47.2%, and Kagoshima = 44.1%), although Fukuoka (13.6%) and Nagasaki (15.0%) showed low capital concentration as well (Fig. 2).

### Okinawa prefecture’s medical care delivery system

Since Okinawa combined the highest retention rate with a distinctive distribution pattern, we undertook a secondary-source-based contextual interpretation of Okinawa’s medical care delivery system using the review-derived domains. This component is presented as interpretive and hypothesis-generating; it does not constitute direct empirical evidence that the described features caused higher retention. Okinawa prefecture’s medical care delivery system has several contextual features that may help interpret the observed pattern when compared with other Kyushu prefectures.

Okinawa Chubu Hospital provides systematic clinical supervision to island physicians through regular clinical guidance and quarterly on-site visits during the nine-year obligatory service [21]. This structured supervisory arrangement mitigates the professional isolation inherent to single-physician remote postings. This mentorship model operates through structured partnerships between Okinawa Chubu Hospital and Okinawa Nanbu Medical Center, as the primary parent hospitals, supporting physicians dispatched to the 16 prefectural remote island clinics [21] (see Additional File 1 for detailed descriptions). Within the review-derived framework, this mentorship and referral structure was interpreted as an institutional support and training arrangement analogous to the professional support and rural rotation mechanisms described in prior physician-retention literature.

Moreover, medical demand is geographically distributed, with clinics established on islands with populations of ≥ 300, resulting in 16 remote island clinics across the prefecture [21, 23]. Compared to several Kyushu prefectures with higher capital concentration, Okinawa showed a more balanced public-private employment profile among retained JMU graduates (32 public vs. 24 private physicians; Table 1).

These structured systems are complemented by additional support mechanisms, including doctor helicopter services and the Yuimaru Project [18], a prefectural substitute physician dispatch and physician recruitment programme operated by the Japan Association for Development of Community Medicine (JADECOM), Okinawa. It mobilises approximately 200 substitute physician days annually to support medical care on remote islands. Within the same framework, these mechanisms were interpreted as institutional supports that may reduce the operational burden of remote-island practice. A conceptual stakeholder and system map summarising these relationships is provided as Figure S1 in Additional File 2. Together, these features may help interpret why Okinawa achieved higher retention than prefectures such as Saga, where capital concentration was high (52.9%) and retention was low (44.2%).

## Discussion

This descriptive comparative study found that Okinawa prefecture had the highest post-obligation physician retention rate (84.2%) among the eight Kyushu prefectures. In this descriptive comparison, geographical isolation alone did not appear sufficient to account for this pattern. No clear linear association was observed between the number of inhabited islands and retention rates (*r* = 0.146, 95% CI = -0.623 to 0.771; *p* = 0.730). However, this ecological comparison was based on only eight prefectures and should be interpreted cautiously.

Okinawa’s comparatively high retention may be interpreted in relation to three contextual domains: (1) comprehensive structured training and support, including systematic clinical supervision, a nine-year rotation programme and substitute physician dispatch through the Yuimaru Project [18]; (2) strategic healthcare infrastructure distribution, left acterised by relatively low prefectural capital concentration (17.2%), 16 remote island clinics, and a balanced public-private employment profile; and (3) cultural cohesion leftacterised by communal reciprocity (*yuimaru*) and a strong regional identity (Additional File 1). These contextual interpretations align with review-based retention factors such as professional support, working environment, rural rotation exposure, and community integration. However, they should be read as hypothesis-generating interpretations rather than empirically verified explanatory factors.

These contextual interpretations are broadly consistent with previous research showing that obligation-based placement alone was insufficient to secure long-term retention [9, 19]. Okinawa’s retention rate exceeded the national JMU average (69.8%), whereas Saga (44.2%) and Nagasaki (46.0%) remained well below this benchmark despite the same obligation framework. By comparing prefectures operating under a shared JMU scheme, the present study extends the literature by showing that substantial retention variation can persist even when the basic bonded-service policy is held broadly constant, thereby highlighting the potential importance of implementation context, supportive institutional arrangements, and local training environments.

Recent Japanese research has similarly highlighted the role of institutional support systems in regional physician retention [14], while international reviews have emphasised community integration, professional development, mentorship, and context-tailored interventions as key determinants of rural retention [2, 5–7, 15]. However, the Japanese setting warrants careful interpretation. Although this study assessed retention among graduates who had completed the obligation period, current practice location could not distinguish voluntary long-term commitment from retention shaped by the long-term influence of the obligation-based scholarship system on career formation and place attachment [9, 20]. Accordingly, the present contextual interpretation should not be understood as direct evidence that any single factor caused Okinawa’s higher retention.

An exploratory observation in this study concerns the relationship between prefectural capital concentration and retention. Okinawa combined relatively low prefectural capital concentration (17.2%) with the highest retention rate (84.2%), whereas Saga showed high capital concentration (52.9%) despite the lowest retention rate (44.2%). However, this pattern should not be overinterpreted, given the small prefecture-level sample sizes inherent in the study design (e.g. Saga: *n* = 34; Okinawa: *n* = 64). Fukuoka (13.6%) and Nagasaki (15.0%) also showed low capital concentration, albeit for different structural reasons: Fukuoka because of its multi-polar urban configuration and Nagasaki because physicians are geographically dispersed across its many inhabited islands. Taken together, these observations suggest that prefectural capital concentration metrics require careful contextualisation. Although high concentration in urban facilities may facilitate out-migration, low concentration does not necessarily indicate effective rural distribution. Hence, these hypotheses merit further testing in studies with larger samples and urban–rural disaggregated analyses.

This study has some important limitations. First, the cross-sectional comparison precluded causal inference. Hence, we could not determine whether institutional support, cultural factors, economic constraints, or other unmeasured regional characteristics were the primary drivers of the observed differences in retention. Second, our empirical analysis was limited to directory-based retention status, whereas the institutional and cultural interpretation relied on secondary sources and contextual interpretation; therefore, the proposed domains remain interpretive hypotheses rather than empirically verified explanatory factors. Third, although this study assessed retention among graduates who had generally completed the nine-year obligation period, current practice location does not distinguish voluntary long-term commitment from retention influenced by the long-term effects of the obligation-based scholarship system and related career pathways. Fourth, key confounders—including spousal employment opportunities, income differentials, working conditions, family considerations, and individual career preferences—were unavailable and may account for part of the observed inter-prefectural variation. Fifth, the study was limited to eight Kyushu prefectures; therefore, generalisability to other regions of Japan and other national contexts remains uncertain. Sixth, the contextual domains were informed by secondary literature rather than primary qualitative or mixed-methods evidence; interview-based studies with physicians, programme administrators, and local stakeholders would be needed to test these proposed mechanisms directly. In addition, the Okinawa component was not designed as a formal qualitative case study with prespecified case boundaries, explicit source-selection criteria beyond the referenced materials, or a reproducible coding procedure. Therefore, it should be interpreted as a structured contextual reading of secondary sources rather than a fully specified contextual analysis. Additionally, geographical barriers to healthcare access were measured using the number of inhabited islands as a proxy, which may not fully capture transportation conditions, distance, weather-related access constraints, or inter-island connectivity. Finally, graduates with unknown practice locations (*n* = 42, 6.6%) were classified as non-retained in the primary analysis. However, the sensitivity analysis showed a similar overall pattern, with Okinawa remaining the highest retention prefecture (Supplementary Table S2).

Despite these limitations, the study contributes a within-programme comparison showing that marked retention variation can persist even under a shared JMU obligation scheme, thereby identifying a policy-relevant research gap. Okinawa’s pattern highlights several hypothesis-generating considerations for rural physician retention. These include deliberately dispersing physician career pathways away from prefectural capitals, providing structured supervisory and support networks that extend beyond the initial obligation period, and aligning programme design with local community values and regional identity. Beyond postgraduate support, these contextual observations may also have implications for medical education reform. Evidence from remote-island and rural retention reviews suggests that island or rural background, early community-based medical education, longitudinal place-based training, and mentorship may strengthen later recruitment and retention [6, 7, 22]. In that context, the Okinawa model suggests that undergraduate and residency curricula may benefit from earlier exposure to community-oriented practice, mentorship by physicians with remote-island experience, and longitudinal ties to local health systems. Such reforms require prospective evaluation, and future interview-based and longitudinal studies are needed to determine whether these proposed mechanisms operate as expected in Okinawa and other settings.

## Conclusion

Okinawa’s comparatively high physician retention may be interpreted in relation to an integrated programme and system context—particularly sustained supervisory and support networks, distributed career pathways, and deliberate decentralisation of training and practice opportunities—rather than geographic isolation alone. Because this study combined a directory-based cross-sectional comparison with literature-based contextual interpretation, these findings should be treated as hypothesis-generating rather than explanatory. Nevertheless, the Okinawa comparison suggests that obligation-based placement may be strengthened by longitudinal support, locally embedded training, and community-aligned programme design. Future research should use longitudinal, interview-based, and multi-regional designs to examine physician motivation, family factors, working conditions, and the long-term influence of the scholarship obligation system.

## Supplementary Information

Supplementary Material 1.

Supplementary Material 2.

Supplementary Material 3.

## Data Availability

The data that support the findings of this study are not publicly available due to confidentiality concerns related to individual physician data from the JMU Graduate Directory, which could compromise participant privacy. Aggregated data are available from the corresponding author upon reasonable request and with permission from JMU.

## Abbreviations

JMU: Jichi Medical University
JADECOM: Japan Association for Development of Community Medicine
