# Supplementary material for: Post-obligation physician retention among Jichi Medical University graduates in Okinawa prefecture, Japan: a descriptive comparative study of the Kyushu region
Source: BMC Prim Care. 2026 Aug 13;27:407. doi: 10.1186/s12875-026-03521-0 (PMC13757055; doi:10.1186/s12875-026-03521-0)
Supplement: Supplementary file 1 [file 12875_2026_3521_MOESM1_ESM.docx]

**Additional File 1**

**Institutional and cultural features that may help interpret physician retention in Okinawa prefecture**

Okinawa prefecture’s high physician retention rate (84.2%) may be interpreted in relation to a combination of institutional support systems and cultural cohesion, which may help contextualise how the prefecture differed from other Kyushu regions.

**Structured training and support systems**

Okinawa’s retention pattern may be interpreted in relation to comprehensive, structured training and support systems, rather than understood primarily through external administrative programs alone.

- **Peer-based mentorship network:** The system functions through experienced clinical mentors who provide practical guidance grounded in field experience. These mentors include senior clinical supervisors from Okinawa Chubu Hospital and Okinawa Nanbu Medical Center, along with former remote island clinic physicians who bring first-hand experience of rural practice [21].
- **Comprehensive nine-year rotation programme:** This systematic rotation provides exposure to diverse practice environments through structured partnerships between Okinawa Chubu Hospital and Okinawa Nanbu Medical Center as primary parent hospitals, and 16 affiliated prefectural remote island clinics. Such arrangements may be relevant to retention, consistent with previous literature on obligation-based training systems [19, 20].
- **The Yuimaru Project:** As a complementary support mechanism, this project provides substitute physician dispatch (approximately 200 physician days annually) and physician recruitment for remote island medical support, working alongside doctor-helicopter services in the context of healthcare delivery across Okinawa’s 38 inhabited islands [18].

**Cultural and social factors**

These institutional systems coexist with several cultural and social features that may be relevant to long-term retention, aligning with international discussions of multi-faceted retention strategies [4, 5].

- **Spirit of *yuimaru*:** Okinawa’s traditional concept of mutual assistance and community support extends to healthcare settings and may provide a cultural context for peer-based mentorship networks. This cultural alignment may complement formal institutional support structures.
- **Strong regional identity:** Okinawa’s unique cultural and historical background is associated with a strong regional identity and attachment among citizens, including medical professionals. Many students from Okinawa entering JMU express a strong commitment to ‘support healthcare in their hometown of Okinawa’, reflecting deep dedication to regional welfare. Recent research in Japan has suggested that such regional attachment may be relevant to physician retention [14].
- **Historical commitment to community healthcare:** This dedication aligns with Okinawa’s post-war establishment of a unique island-adapted healthcare system featuring public health nurses and medical assistants stationed on remote islands [21]. These institutional frameworks have been sustained and refined over decades, with ongoing policy efforts to protect health services across Okinawa’s remote islands [21], demonstrating a long-standing institutional and cultural commitment to addressing geographical healthcare challenges.

**Practice environment**

JMU graduates’ working environments in Okinawa prefecture showed a relatively balanced distribution across different practice settings; this pattern may be considered alongside the presence of structured support systems (Table 1).

- **Public–private balance:** Among the JMU graduates who completed their obligation period, 32 worked in public hospitals or clinics, 24 worked in private hospitals, and 1 worked at a university hospital, indicating diverse career opportunities without marked concentration in a single sector.
- **Geographical distribution:** Among the physicians, 11 were based in Naha city (17.2%), with 53 working outside the prefectural capital, suggesting a relatively broad distribution across the prefecture. This contrasts with most other Kyushu prefectures, where capital concentration exceeds 30% (Kumamoto 61.4%, Saga 52.9%), except Fukuoka (13.6%) and Nagasaki (15.0%), whose low concentrations reflect structurally different configurations rather than deliberate rural dispersal (see main text for details).
- **Career path diversity:** In addition to hospital physicians, six JMU graduates entered private practice, whereas one graduate worked in public health administration, highlighting the diverse career opportunities available within the prefecture in the presence of structured training and mentorship networks.

**Comparison with other Kyushu prefectures**

Okinawa’s approach can be interpreted more clearly when compared with other prefectures in the region. Despite Saga recording the second-highest prefectural capital physician concentration in the region (52.9%), its retention rate (44.2%) remains the lowest. This pattern may help contextualise how high urban physician concentration, when not balanced with community-based career pathways and peer mentorship, could coincide with out-migration. Similarly, while Nagasaki has more inhabited islands (51) than Okinawa (38), its retention rate (46.0%) is substantially lower, suggesting that island number alone may be insufficient to interpret retention patterns across prefectures. However, economic considerations may also be relevant to these patterns, as graduates face substantial financial penalties (approximately ¥22.6 million) if they fail to complete their nine-year obligation [9, 20].
