# Supplementary material for: Post-obligation physician retention among Jichi Medical University graduates in Okinawa prefecture, Japan: a descriptive comparative study of the Kyushu region
Source: BMC Prim Care. 2026 Aug 13;27:407. doi: 10.1186/s12875-026-03521-0 (PMC13757055; doi:10.1186/s12875-026-03521-0)
Supplement: Supplementary file 2 [file 12875_2026_3521_MOESM2_ESM.docx]

**Additional File 2**

**Conceptual stakeholder and system map of Okinawa’s physician-retention support system**

This conceptual figure illustrates the relationships among the JMU policy framework, the two parent hospitals, the 16 remote island clinics, JADECOM Okinawa’s Yuimaru Project, emergency support mechanisms and the cultural foundation of *yuimaru*. It is a descriptive systems map rather than an empirical causal model.

Figure S1. Conceptual stakeholder and system map of the Okinawa model. Parent hospitals, substitute physician dispatch, emergency transfer support, and community culture may help contextualise professional isolation and distributed career pathways and may be relevant to higher post-obligation retention.
