# Supplementary material for: Post-obligation physician retention among Jichi Medical University graduates in Okinawa prefecture, Japan: a descriptive comparative study of the Kyushu region
Source: BMC Prim Care. 2026 Aug 13;27:407. doi: 10.1186/s12875-026-03521-0 (PMC13757055; doi:10.1186/s12875-026-03521-0)
Supplement: Supplementary file 3 [file 12875_2026_3521_MOESM3_ESM.docx]

**Supplementary Table S2. Sensitivity Analysis: Retention Rates with and without Unknown Cases**

This table presents a comparison of physician retention rates calculated according to (1) a conservative approach, with unknown workplace locations classified as non-retained (primary analysis), and (2) an alternative approach, with unknown cases excluded from the denominator (sensitivity analysis).

| **Prefecture** | **Total Graduates (n)** | **Unknown Cases (n)** | **Retained in Prefecture (n)** | **Primary Analysisᵃ (%)** | **Sensitivity Analysisᵇ (%)** | **Difference (%)** |
| --- | --- | --- | --- | --- | --- | --- |
| **Okinawa** | 76 | 5 | 64 | 84.2 | 90.1 | +5.9 |
| **Kagoshima** | 87 | 2 | 59 | 67.8 | 69.4 | +1.6 |
| **Oita** | 81 | 10 | 53 | 65.4 | 74.6 | +9.2 |
| **Miyazaki** | 82 | 2 | 50 | 61.0 | 62.5 | +1.5 |
| **Fukuoka** | 75 | 8 | 44 | 58.7 | 65.7 | +7.0 |
| **Kumamoto** | 76 | 5 | 44 | 57.9 | 62.0 | +4.1 |
| **Nagasaki** | 87 | 6 | 40 | 46.0 | 49.4 | +3.4 |
| **Saga** | 77 | 4 | 34 | 44.2 | 46.6 | +2.4 |
| **Total** | **641** | **42** | **388** | **60.5** | **64.8** | **+4.2** |

**Notes:** ᵃ Primary Analysis: Retention rate calculated with unknown workplace locations (n = 42, 6.6%) classified as ‘non-retained’ (conservative approach). Denominator = all graduates (n = 641).

ᵇ Sensitivity Analysis: Retention rate calculated with unknown workplace locations excluded from the denominator. Denominator = graduates with known workplace locations (n = 599).

**Key Findings:**

1. Overall pattern remained similar across analyses: Okinawa demonstrated the highest retention rate in both approaches (84.2% vs. 90.1%). Although some prefectural ranks changed modestly when unknown cases were excluded, the main interpretation of Okinawa as the clear outlier in retention did not change.

2. Modest overall impact: Excluding unknown cases increased the overall retention rate by 4.2 percentage points (60.5% → 64.8%), indicating that the conservative classification of unknown cases as ‘non-retained’ did not substantially alter the study’s main conclusions.

3. Prefecture-specific differences: The impact of unknown cases varied across prefectures, with the largest impact in Oita (+9.2 percentage points) and the smallest in Miyazaki (+1.5 percentage points) and Kagoshima (+1.6 percentage points).

4. Robustness of the main finding: Okinawa’s exceptionally high retention rate remained substantially higher than the overall average and all other prefectures in both analyses, supporting the study’s primary conclusion.

**Interpretation:**

The sensitivity analysis demonstrates that the conservative approach of classifying unknown workplace locations as ‘non-retained’ did not materially affect the study’s main conclusion regarding Okinawa’s exceptionally high retention rate. However, the analysis highlights the importance of minimising unknown cases in future studies, particularly in prefectures with higher proportions of unknown locations.

*Abbreviations: n = total number*
